# Speeding Task Allocation Search for Reconfigurations in Adaptive Distributed Embedded Systems Using Deep Reinforcement Learning

**DOI:** 10.3390/s23010548

**Published:** 2023-01-03

**Authors:** Ramón Rotaeche, Alberto Ballesteros, Julián Proenza

**Affiliations:** Departament de Matemàtiques i Informàtica, Universitat Illes Balears, 07122 Palma de Mallorca, Spain

**Keywords:** Deep Reinforcement Learning, Distributed Embedded Systems, combinatorial optimization, Machine Learning

## Abstract

A Critical Adaptive Distributed Embedded System (CADES) is a group of interconnected nodes that must carry out a set of tasks to achieve a common goal, while fulfilling several requirements associated with their *critical* (e.g., hard real-time requirements) and *adaptive* nature. In these systems, a key challenge is to solve, in a timely manner, the combinatorial optimization problem involved in finding the best way to allocate the tasks to the available nodes (i.e., *the task allocation*) taking into account aspects such as the computational costs of the tasks and the computational capacity of the nodes. This problem is not trivial and there is no known polynomial time algorithm to find the optimal solution. Several studies have proposed Deep Reinforcement Learning (DRL) approaches to solve combinatorial optimization problems and, in this work, we explore the application of such approaches to the task allocation problem in CADESs. We first discuss the potential advantages of using a DRL-based approach over several heuristic-based approaches to allocate tasks in CADESs and we then demonstrate how a DRL-based approach can achieve similar results for the best performing heuristic in terms of optimality of the allocation, while requiring less time to generate such allocation.

## 1. Introduction

A Distributed Embedded System (DES) is a combination of hardware and software, where the hardware is a set of interconnected *nodes* and the software is typically implemented as a set of computational elements, called *tasks*, which are executed in the nodes in order to achieve some common goal. DESs play a key role and are almost ubiquitous in many engineering fields, such as avionics, automotive industry, healthcare, energy management or telecommunications.

DESs are used in real-world applications and some of them have *real-time* (RT) and *dependability* requirements. We refer to DESs that have demanding RT and dependability requirements (i.e., strict real-time response and very high dependability) as *critical* DESs. A system is said to have RT requirements if its correct operation depends not only on its ability to provide a correct response, but also on its ability to provide such a response before some deadline. On the other hand, a system is said to have dependability requirements [1] if it is required to have certain attributes that provide enough trustworthiness in the system’s ability to provide a correct service.

**Figure 1:**
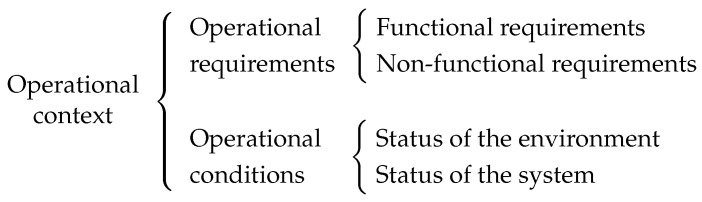
Components of the operational context.

Nowadays there is strong interest in using critical DESs in changing *operational contexts*. By operational context, we mean all the relevant aspects involved in the operation of the system that are susceptible to change. As seen in Figure 1, the operational context includes the functional requirements (i.e., the fundamental functionalities the system must carry out) and the non-functional requirements (i.e., the RT guarantees and the dependability guarantees). All these requirements are referred to as *operational requirements*. Additionally, the operational context also considers the *operational conditions*, that is, the circumstances under which the system has to operate. These are the state of the environment and the state of the system itself which could change, for example, due to faults in its components. For a critical DES to operate efficiently and effectively under unpredictable changes in the operational context, it needs to be *adaptive* [2]. Adaptivity, in the context of a Critical Adaptive Distributed Embedded System, implies that the system must be able to dynamically assign its computing and communication resources while in operation, in response these kind of changes.

A Critical Adaptive Distributed Embedded System (CADES) includes a software component we call Node Manager (NM) that continuously monitors the operational context and, if a change jeopardizes the ability of the system to operate correctly, finds and applies a new proper *system configuration*. A system configuration (or *configuration* for short) determines the allocation of tasks and messages to nodes and network links, respectively, as well as all their RT and reliability attributes. On the one hand, RT tasks and messages have schedule-related attributes like periods, deadlines or priority levels. On the other hand, if the tasks and messages have stringent dependability requirements, their level of replication must be defined. Replication consists in executing redundantly (multiple times) a given task or a given communication in order to tolerate faults affecting some of the replicas. Finally, note that the longer it takes to reconfigure the system, the longer the system is not providing the expected services and thus the reconfiguration time must be short. Moreover, since tasks in CADES can have RT requirements, it is desirable that the reconfiguration is also carried out in a timely manner.

The first and most important aspect in a configuration is finding a task allocation in which the RT requirements of all the tasks are met. This is because the rest of the aspects, such as the communications or the replication of the tasks, explicitly depend on where each task is placed. An allocation of RT tasks must guarantee, in each node, that the tasks being executed meet their deadlines. That is, the NM must not only distribute the tasks, but perform a schedulability analysis in each of the nodes. In this regard, we recommend using a utilization-based schedulability test. As further explained in Section 2.4, in [3] the authors demonstrated that it is possible to guarantee that the tasks being executed in a node are schedulable if the sum of their utilization of the CPU is below a predefined threshold. Note that the utilization of a task can be easily calculated from its period and execution time. This approach makes it possible for the NM to validate the RT response of a configuration in a short time, contrary to typical schedulability tests which operate offline.

The problem can, then, be formulated as follows. Given the set $T=\{u_{1},u_{2},\cdots ,u_{n}\}$, where $u_{i}$ is the utilization of task *i* and a set of *m* nodes, each one with the same capacity, assign all the tasks to the nodes so that, for each node, the sum of the utilizations of the tasks being executed in said node does not surpass a certain predefined threshold. Note that here we assume that all the nodes are identical, they all have the same capacity.

There are many criteria that can be followed to decide on the task allocation. However, we believe that assigning all the tasks to the minimum number of nodes is preferable. The main advantage of this approach is that it minimizes the number of nodes the system must be provisioned with to correctly operate. Consequently, it reduces the cost, weight, size and energy consumption, which is desirable in an embedded system. Later, in Section 2.3, we list and discuss all the advantages of this approach. If this criterion is selected, the problem is then equivalent to the well known bin packing problem (BPP) [4,5]. If we formulate the problem in terms of the BPP, tasks are *items*, utilizations are *sizes* and nodes are *bins*.

BPP is an NP-hard combinational optimization problem, meaning that there is no known polynomial time algorithm to find the optimal solution [6]. These types of problems are typically solved using approximation algorithms, which provide near-optimal solutions with a low time complexity. These algorithms can be classified into *online* and *offline*. Online algorithms sequentially assign items to bins without considering any item that has not been packet yet. That is, the bin to which item *i* is assigned only depends on the items [1,i]. Offline algorithms, in contrast, consider the complete list of items to perform the assignment. The main advantages of online algorithms are that they do not require a previous knowledge of the list of items and that they require less computation time. In contrast, offline algorithms provide better approximations.

Later, in Section 5.1 we explain some of the most utilized online and offline approximation algorithms. In particular, first-fit-decreasing (FFD) is a well known offline algorithm used as the reference in many BBP-related works [4,7,8,9,10]. This is because FFD provides solutions very close to the optimal in a relatively short execution time. Specifically, FFD produces, in the worst case, a solution with a number of bins equal to $\frac{11}{9}OPT\left(L\right)+\frac{6}{9}$ (with *OPT*(*L*) being the minimum number of bins necessary to pack items in list *L*) [11] with a time complexity of O(|L|log(|L|)) (with |L| being the number of items in list *L*).

Note, however, that in an adaptive system it is desirable that the new configuration is determined as soon as possible. This is because, when a change in the operational context occurs, the system stops providing its intended service until the new configuration is applied. Moreover, if we think about the dependability, if a change in the operational context jeopardizes the ability of the system to provide the required fault tolerance, during this time, the system is more vulnerable to faults.

Several studies have shown that Machine Learning (ML), in general, and Deep Reinforcement Learning (DRL), in particular, can outperform other approaches in solving combinational problems like the one previously presented. The goal of this work is to demonstrate that DRL techniques can be used to find new system configurations in CADESs and provide good results in terms of optimality while requiring less time to generate such results. For this, we design and implement a DRL-based approach capable of allocating tasks to nodes in a CADES and analyze the benefits of such an approach both qualitatively and quantitatively. In order to achieve such an objective, we executed the following tasks:

1. Define the task allocation problem to solve (e.g., allocation criteria, assumptions made);
2. Select the best ML-based approach to solve the problem;
3. Analyze the potential benefits of the selected approach qualitatively;
4. Define and implement the selected approach;
5. Execute the experiments to quantitatively analyze the benefits of our approach in comparison with typical approaches used in this kind of problem.

In a previous four-page work-in-progress [12], we identified the potential benefits of using DRL to solve combinational optimization problems like the allocation of tasks in CADES. Specifically, we designed, implemented and tested a preliminary version of a DRL-based solution to allocate tasks in a CADES. In the present paper, we extend the work carried out:

- We give a detailed explanation of the complete problem of finding configurations in CADES and, then, of the specific aspects of this problem that we cover here.
- We give a detailed explanation of the assumptions made to ensure that the solutions we provide meet the RT requirements.
- We give a detailed description and motivation of the specific Machine Learning techniques used in our DRL model.
- We give a detailed description of the inputs, outputs, architecture and training process used to build and train our DRL model.
- We modify the design to introduce a new manner of assigning tasks to nodes.
- We include new experiments to test with a bigger and variable-size sets of tasks, as well as to measure the response time in a more comprehensive manner.
- We discuss in more detail the further work suggestions.

The remainder of this document is structured as follows. In the next section, we specify the problem to be solved, including the necessary assumptions and simplifications. In Section 3 we justify the use of a DRL-based approach and introduce the theory behind DRL and the specific techniques employed in this work. In Section 4, we present our DRL-based solution. In Section 5, we show the experiments we have carried out together with their results. Additionally we compare these results with those obtained for heuristic-based solutions. In Section 6 we discuss related work. Finally, in Section 7, we discuss the conclusions and future work.

The remaining sections assume some basic understanding of ML concepts and, in particular, of the design and training of Deep Neural Networks (DNNs).

## 2. Problem Statement

As with many engineering and mathematical problems, a reasonable approach to solving a complex issue is to break it down into smaller and simpler parts on which to gradually build a solution. In line with this principle, we decided to narrow the scope of this work by simplifying the concepts of system configuration and operational context introduced in the previous section.

### 2.1. System Configuration

In this work, we focus on a key aspect of a system configuration: the *task allocation*. Given a set of tasks that must be executed, a *task allocation* can be defined as the distribution of such tasks into the nodes of the system. More formally, it is a many-to-one binary relation between the set of tasks and the set of nodes available.

We leave aside other elements of the system configuration such as the task replication or the communications between nodes. Therefore, in the rest of this document, we use the term “task allocation” rather than “configuration” to emphasize that we are only solving this aspect of the system configuration. Similarly, we refer to “task reallocation” rather than “reconfiguration” when talking about the specific act of finding a new task allocation after changes in the operational requirements.

It must be noted that the ability to reallocate tasks not only makes the system more adaptive, but also contributes to improving its dependability. This is because such reallocation ability allows the system to recover tasks, meaning that it can tolerate faults affecting tasks or nodes.

### 2.2. Modelling Tasks and Nodes

As discussed in the introduction, Adaptive Distributed Embedded Systems must be able to operate under unpredictable dynamic *operational contexts*, which encompass both the operational requirements and the operational conditions.

The operational requirements considered in our problem are the set of tasks that must be allocated. We assign a *computational cost* to each task, which is a scalar number representing the quantity of resources that each tasks needs in order to be properly executed.

The operational conditions considered in our problem are the resources available to execute tasks in each node, which we refer to as the *computational capacity* of each node. A node’s computational capacity determines the amount of available resources. The tasks allocated to a given node cannot add up to a total computational cost higher than the node’s capacity. To simplify, we consider that the computational capacity is constant for all the nodes and throughout the entire duration of the system operation.

The reason for modelling the system in terms of computational costs and computational capacities is because, as will be explained in Section 2.4, we can apply a utilization-based test to check the RT response of the system. Specifically, if the Node Manager (NM) is able to allocate all the tasks to the available nodes so that no node receives a subset of tasks with an aggregated computational cost higher than its computational capacity, we can guarantee that the tasks will meet their deadlines. That is an easy and fast manner of checking the RT response of a given task allocation.

Finally, we assume that a change in the operational requirements triggers a change in the list of tasks to be executed. This is in contrast to a non-adaptive system where the NM would only be programmed to deal with some specific sets of tasks defined a priori.

### 2.3. Task Allocation Criteria

**Figure 2:**
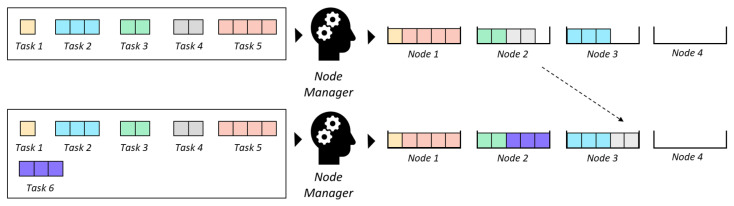
Minimizing the number of active nodes—Illustrative example. A reallocation in order to keep the number of active nodes at a minimum.

When the set of tasks that must be allocated changes (i.e., when the operational requirements change) the NM must find a new allocation of tasks. Such allocation could be chosen based on different criteria. We will model the problem where the NM must find the task allocation that, for a given set of tasks, the minimum number of nodes is used, which we will refer as the *number of active nodes*. See the example in Figure 2: initially, the set of tasks to be allocated is tasks 1 to 5 (represented in the top half of the picture), but if in a later step a new task (Task 6 in the picture) needs to be allocated, the new set of tasks that the NM must allocate is tasks 1 to 6, which triggers a reallocation.

Minimizing the number of nodes is preferred over other criteria for various reasons:

- The system can be designed to include a restricted number of nodes, which reduces its cost, weight and size.
- At runtime, the number of active nodes is the minimum and, thus, the energy consumed by the nodes is also minimized.
- Concentrating tasks reduces the network traffic as tasks executed in the same node can exchange messages without using the communication channel. This simplifies the scheduling of the RT messages.
- From a research point of view, the problem is equivalent to the bin-packing problem, which means there are multiple algorithms already available that can be compared with in terms of the performance.

Of course, concentrating tasks in a minimum number of nodes has its own disadvantages (e.g., more challenging scheduling in each of the active nodes, higher severity in the event of a failure) and therefore it is not our intention to present this allocation criteria as a sufficient condition for a well designed CADES.

It must be noted that, although we model the search for task allocations that minimizes the number of nodes as an optimization problem, our system does not need to find the *best* possible task allocation (i.e., the optimum). The system can work with just "good" task allocations. In fact, our proposed approach will find good solutions but is not guaranteed to find the optimal solution. This is completely expected since, as discussed later, no algorithm has been found for this problem, other than an exhaustive search, capable of guaranteeing the finding of the optimal solution for any given set of tasks.

### 2.4. Assumptions Made

We consider the problem in which all the tasks that are required for a correct operation of the system can be allocated to the available nodes. Therefore, it is necessary to guarantee the existence of an allocation that includes *all* the tasks (i.e., no task is left without a node assigned). This could be enforced in the design of a real system by having a number of nodes large enough to guarantee a solution given the maximum possible number of tasks and the possible maximum cost. In a real scenario, this cannot be fully guaranteed because there is always the possibility of failing nodes, meaning there is always a chance that there are not enough nodes to execute all tasks. In this work, we will assume that in such a case, we would have some mechanism by which the system enters in *degraded mode*, taking measures such as prioritizing the most critical tasks or stopping replicating some tasks.

An additional condition is required to meet the RT requirements, which is that the subset of tasks assigned to each node is *schedulable*. Schedulable means that, given each task’s computation time and period, it is possible to find a schedule for the node that always meets the deadlines for said tasks.This requirement could be enforced (for nodes that use RT operating systems applying rate-monotonic scheduling) by capping each of the nodes’ capacities made available in the allocation process to 69.3% of its *actual* computational capacity, since *Liu & Layland* [3] proved that for any set of *n* periodic tasks, a schedule exists if the resulting node’s utilization is below 69.3%. The node’s utilization is the sum of each task’s utilization, which is defined as the computation time required to execute the given task in the given node, divided by the task’s period. Therefore, in order to use this theorem to enforce our schedulability assumption (i.e., that the subset of tasks assigned to each node is schedulable) in practice, it would require expressing the tasks’ computational cost as the number of operations required each time the task is executed divided by the task’s period and expressing the node’s computational capacity as 0.693 multiplied by the number of operations that the node can execute in a time unit.

### 2.5. Resource Requirements

Lastly, two requirements related to the task reallocation that are common in CADESs have been considered: memory requirements and latency. Given that in many systems the software implementing the NM needs to be deployed in resource-constrained processors such as microcontroller units (MCU), it is important to develop an algorithm that can be stored in these devices’ flash memory, which is up to 1MB in industrial settings [13], and that is able to generate a new task allocation as fast as possible to increase the likelihood that not only the tasks meet their RT requirements but also that the reconfiguration itself can be done in a quick manner (although we will not try to establish any strict RT response requirements for the reconfiguration itself). In our work, we have factored these requirements by ensuring that our model’s size is below 1MB and by having the inference latency as one of the dimensions benchmarked in our experiments.

### 2.6. Summarized Problem Statement

Recapitulating, the actual problem tackled in this work has been the design of a solution capable of allocating tasks to nodes ensuring that, for each node, the sum of the costs of its assigned tasks does not surpasses is capacity while, at the same time the total number of active nodes (i.e., nodes that receive at least one task) is minimized. In addition, special attention will be paid to the memory requirements and time necessary to produce a solution.

## 3. Introduction to DRL and Motivation for a DRL-Based Approach

The problem we are trying to solve (as described in the previous section) is an optimization problem, since we want to minimize the number of active nodes. More precisely, it is a *combinatorial optimization* problem, which is a type of optimization problem where the objective is to find the optimal object from a finite set of objects (in our case, the set of objects would be the set of all possible ways in which the tasks can be allocated and an object would be a specific mapping between tasks and nodes).

As mentioned in the previous section, our problem is equivalent to the bin-packing problem [4], which is an NP-optimization problem, meaning that there is no known polynomial time algorithm to find the optimal solution [14]. These types of problems are typically solved using solvers (e.g., [15]) or heuristics. In this work, we wanted to explore alternative approaches based on ML techniques, which, as detailed later in this section, might present a number of advantages.

In the rest of the section, we first introduce the concept of Deep Neural Network (DNN), which is a key component of the discussed Machine Learning approaches. Secondly, we introduce the concept of supervised ML and discuss why it is not fit for our purpose. We then introduce the concept of Reinforcement Learning and Deep Reinforcement Learning (DRL). Lastly, we elaborate on why DRL is the right approach for this problem and reflect on its suitability to a CADES.

### 3.1. Deep Neural Networks

As discussed in Section 2, in our problem the input is a set of tasks and the computational cost of each task, while the output must be some representation of how these tasks are assigned to the available nodes. We need to learn some way to map our inputs to the output. Given the high dimensionality of our input–output pairs, we considered Deep Learning (DL) that makes use of *Deep Neural Networks* (DNNs) [16] to parameterize the input–output mapping function.

A deep neural network, given an input *x* and an output *y*, is a mapping function $y^{\prime }=f\left(x,\theta \right)$ that tries to approximate the unknown function $f^{*}$ which maps any possible set of input–output pairs $y=f^{*}\left(x\right)$. The symbol θ represents the parameters that define this mapping function (i.e., the operations that map *x* to *y*) and they can be “learnt" using different ML algorithms. There are many types of DNNs, which differ in the computations they perform to compute the output based on the input. However, all DNNs have some things in common; that is precisely what makes them a DNN.

First of all, the input and outputs are always represented as multidimensional arrays (a.k.a. tensors), with no specific restriction on the number of dimensions. For example, the output can be a simple scalar (which can be seen as a 1 × 1 array) or a colored image, which is represented as a three-dimensional array.

**Figure 3:**
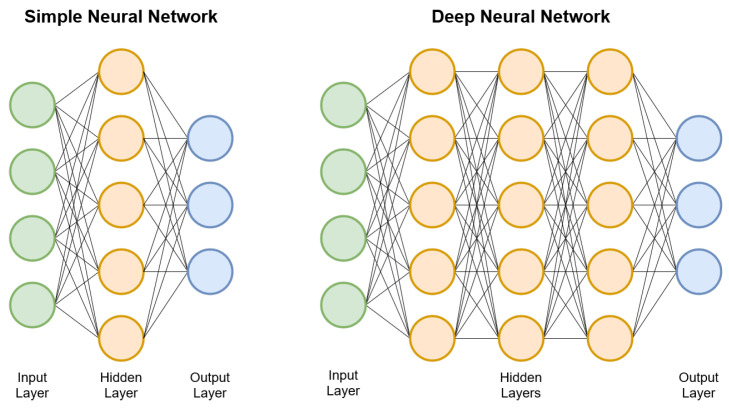
Schematic view of a Neural Network. Deep Neural Networks (DNNs) are just Neural Networks with multiple hidden layers.

Secondly, all the computations of a DNN can be represented as a composition of functions; each function is referred to as a *layer* that takes the array output by the previous layer, performs a mathematical operation and returns a new array. The array taken by the first layer would be the input and the array returned by the last array would be the output. This layered representation is the reason we call it a “network”, and they are called “deep” because they typically chain a large number of layers. See Figure 3.

**Figure 4:**
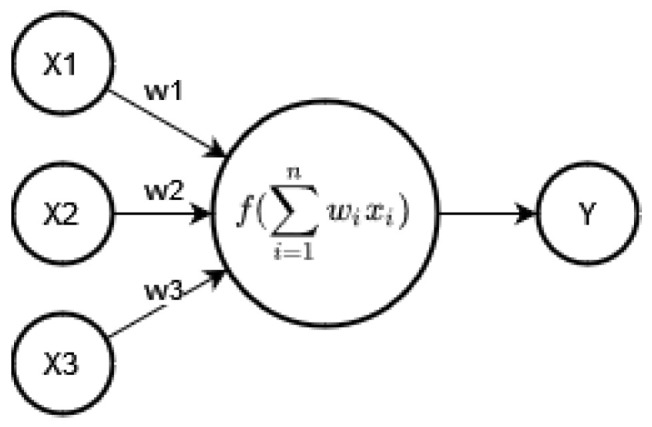
Schematic view of a neuron. Elements from the array returned by the previous layer are multiplied by a scalar (the parameters θ) and then a non-linear activation function is applied.

Lastly (Figure 4), each of these layers typically has several *neurons* which take each element from the input array, multiply it by a scalar and then apply an *activation function*. The scalars that multiply the elements of the input array are precisely the learnt parameters θ of a network. The activation function is typically a non-linear transformation (e.g., ReLU [16], soft-max [16]).

### 3.2. Supervised Machine Learning

The first family of ML techniques that we considered was supervised ML, which allows one to infer a function (a.k.a. learn a function) that is able to map input data to an output (a.k.a. estimation or prediction) even if the model has never ‘seen’ those input data before. That is why it is said that supervised ML models learn to ‘generalize’. In order to do this, supervised ML techniques require a training dataset with examples of input–output pairs. However, supervised ML is quite limited for our purposes, because it requires examples of the optimal task allocation (desired output) for each set of tasks in the training dataset. There is no known polynomial time algorithm to find the optimal solution for our problem [14], so generating optimal solutions is complex and time consuming, especially with large sets of tasks.

### 3.3. Reinforcement Learning

**Figure 5:**
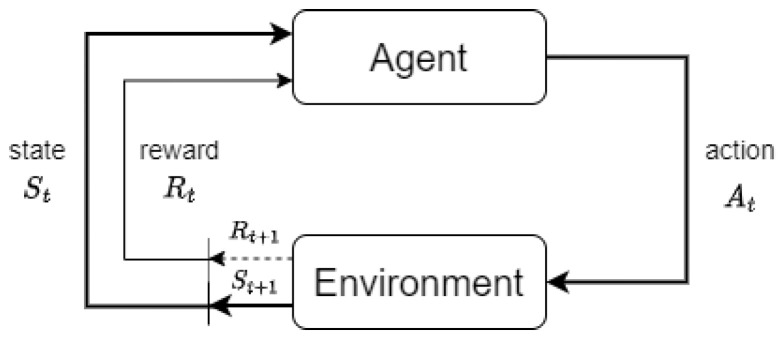
The agent–environment interaction in an RL decision process.

Once supervised learning was discarded for the reasons described in the previous section, further research led us to conclude that the right approach for this problem was Reinforcement Learning (RL). Reinforcement Learning (RL) is a sub-field of Machine Learning that studies methods for a decision maker, the *agent*, to learn what *actions* to take in a given *environment* to maximize a numerical value, the *reward*, accumulated over time [17]. The RL problem can be formalized using the decision process shown in Figure 5, where the agent, each time step *t*, receives the *state* of environment $S_{t}$ and selects an action $A_{t}$ that modifies it. In response to this action, the agent receives a reward $R_{t}$ and the new state $S_{t+1}$.

The criteria used by the agent to decide on the action to take is called *policy*. A policy maps states to actions, which is typically denoted as $\pi (A_{t}|S_{t})$. Sometimes, in solutions based on RL the policy is *parameterized*; that is, the mapping between states and actions is a parameterized function. Here the objective is to learn the parameters (i.e., learn the policy) that makes it possible to score the maximum reward. The value of the parameters, denoted as θ, are updated taking into account the reward obtained in the interactions with the environment. One way to do this is by using the *policy gradient method* [17], which is the technique we used and which we will explain in more detail in the next section.

### 3.4. Deep Reinforcement Learning

One way to parameterize the policy that is followed by the agent is with a DNN, since a DNN is effectively a parameterized function that takes an input (in this case, the state) and produces an output (in this case, the action). Using a DNN is particularly useful when the state space is very large (or infinite), as in our case where the number of different sets of tasks that the agent can receive is very large (as the number of total tasks can vary as well as the cost of each task). Given that we use a DNN to parameterize the policy, our approach falls into the field of Deep Reinforcement Learning (DRL), which is the term used to refer to the set of RL techniques that make use of DNNs. Note that our approach is only one of many ways in which DNNs can be used in RL, so the method we will detail later on is not a comprehensive view of what DRL is.

### 3.5. Rationale for a DRL Approach

One of the main reasons for DRL being a better approach to our problem is that, as opposed to supervised ML, the model can learn to take better actions (in our case the actions would be the task allocation) without actual examples of what the right action is. The only signal it needs is the reward associated with each action that is undertaken. For example, in our case and based on the problem statement from the previous section, the reward would be higher the lower the number of active nodes.

One can see that our problem fits perfectly in the RL framework: the NM (the *agent*) has to select among a set of possible task allocations (the *actions*), based on the tasks cost and nodes capacity (the *state*), in order to minimize the number of active nodes (the *reward*). In fact, although the study of modern RL falls under the ML umbrella, its “modern fathers” A. Barto and Richard S. Sutton [17] were highly influenced (as declared by themselves [17]) by previous ideas in the field of *adaptive* optimal control (e.g., [18]), a field concerned with controlling unpredictable dynamical systems and which one could see as more closely related to CADESs.

Lastly, it is worth mentioning that when we first decided to explore the use of DRL to tackle this problem, although we had some intuitions concerning its potential advantages, it was driven primarily by the desire to undertake such an academic exercise. However, as we progressed in our research, we realized that a DRL-based approach, when compared to solvers and heuristic-based algorithms, could have several advantages that make it a real alternative for CADESs. Namely:

- There are studies proving that DRL-based solutions are near as good or even better, than popular heuristics for solving combinatorial optimization problems [19,20].
- The algorithm used to teach the agent can maximize any *reward* function. Conversely, when solving a combinatorial optimization problem using solvers or heuristics the solution is typically specific to the problem statement. This is particularly significant when the problem involves high-dimensional states, that is, states with a large number of variables and/or complex reward functions. In these cases, building a solution based on heuristics can require non-negligible ad hoc work.
- After the DNN has been trained, the inference latency (i.e., the time it takes to produce a solution) is relatively low. Moreover, compared to many heuristics, it can be lower. This is because heuristics may require exploring the search space, sort the inputs, etc. Furthermore, depending on the specific techniques used, the inference latency can also be bound in time. All of this suggests that a DRL-based approach can be used in CADESs with real-time requirements.
- Although the training of a DNN-based solution can require a non-negligible amount of computational power, complex generated models can be deployed on processors with constrained resources like microcontrollers (MCUs), thanks to Tiny ML [13,21]. Moreover, the concessions carried out to reduce the resource requirements do not significantly impact the inference accuracy and latency. All of this suggests that a DRL-based approach can be used in resource-hungry CADESs.

## 4. Problem Formulation and Approach

As discussed in Section 2, in this work we aim at designing a solution capable of allocating tasks to nodes in a way that, for every node, the sum of the costs of its allocated tasks does not surpasses its capacity. Moreover, we minimize the total number of active nodes (i.e., nodes that receive at least one task).

Our task allocation problem maps to the DRL framework discussed in the previous section as follows:

1. The *state* is the set of tasks, each of them with a cost, that must be allocated;
2. The *environment* is the set of nodes available and its capacity;
3. The *action* is a specific mapping of tasks to nodes;
4. The *reward* must be some scalar that is inversely proportional to the number of nodes used (since we want to minimize this number);
5. The *policy* is a DNN that maps any given state to an action (i.e., any given set of tasks to a specific allocation of tasks to nodes).

Before jumping into the details in the upcoming subsections, let us discuss the items listed above, which will help to outline the design decisions that are required to formulate an approach. These design decisions are then discussed one by one in the upcoming subsections.

If we want our DNN to take a given set of tasks as an input and generate as the output a specific allocation of those tasks to nodes, the first thing we need is a way to numerically *represent* both a set of tasks and an allocation of tasks to nodes. This is what we call the *input–output representation approach* and is discussed in Section 4.1.

The second thing we need to define is how the reward is computed, because as mentioned above, the only real restriction we have is that it needs to be scalar, that is, inversely proportional to the number of nodes used. This, the *reward signal approach*, is what we discuss in Section 4.2.

Third, we need to choose a specific RL technique that can be used to “teach” our policy to allocate tasks in a way that maximizes the reward. For the sake of clarity, note that “teaching our policy to allocate tasks in a way that maximizes the reward” is equivalent to saying “finding the DNN parameters that map inputs to outputs in a way that maximizes the reward”. The *RL approach* is discussed in Section 4.3.

Lastly, the DNN has to take the input (the set of tasks) and generate the output (the allocation of tasks to nodes). As mentioned, the numeric format for the input and the output is discussed in Section 4.1. However, we also need to define the operations that take place to compute the output based on the input, which depends on the architecture of the DNN. This, the DNN’s architecture, is discussed in Section 4.4.

### 4.1. Input–Output Representations Approach

In our parameterized policy (i.e., our DNN), the input (i.e., the state) is the set of tasks that must be allocated. We decided to represent it as a sequence *S* of *l* tasks, each one with an associated cost $c_{i}$, i∈[1,L].

**Figure 6:**
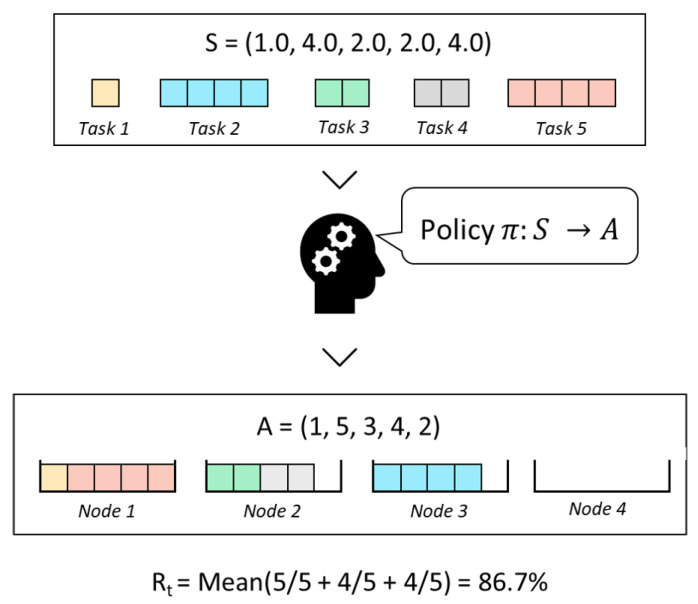
Input–output representation example for a set of five tasks and node capacity C = 5, for the case where the NF allocation heuristic is followed. The set of available tasks is a sequence containing the cost of each task. Concerning the output of the DNN, the sequence A is the order in which the tasks are allocated following the NF rule. This is a simple example, but maximizing the occupancy ratio is an NP-optimization problem, which becomes challenging to solve for large sets of tasks.

We decide to represent a task allocation (i.e., a specific mapping of tasks to nodes) as follows. Our representation (i.e., the output of the DNN) will be a sequence *A* that represents the order in which the tasks in *S* are allocated to nodes following a given heuristic *H*. To represent this allocation order, we define *A* as a sequence of *L* integers $a_{i}\in \left[1,L\right]$, i∈[1,L], where $a_{i}$ indicates the position of the task in the input sequence *S*. This representation is better understood with an example, such as the one shown in (Figure 6).

With regards to the heuristic *H* that we use once we have defined the allocation order *A*, we will experiment with two:

- *Next-Fit* (NF): In this heuristic, tasks are allocated in the first available node until task $a_{i}$ does not fit. At this point, $a_{i}$ is allocated in the next node, which keeps receiving tasks until it can no longer handle the current task, and so on.
- *First-Fit* (FF): In this heuristic, as in NF, tasks are sequentially allocated to the first available node until a given task does not fit. However, when this happens, if the node is not completely full, the node is not “closed". It is kept “open" for future allocations. In each allocation step, “open" nodes are sequentially checked until a node with enough available capacity is found.

Note that what we are doing is using the allocation order sequence *A* generated by the DNN to implicitly dictate how tasks are grouped together. We have also considered other representations for the output. For instance, it would be possible to represent it as the sequence of indexes of the nodes where each of the tasks is allocated. Note, however, that this leads to a large equivalence class of solutions and, as stated in [19], the more restricted the equivalence class for the outputs, the better the results obtained. Moreover, the chosen representation, when combined with the selected allocation heuristic, guarantees that all the solutions produced fulfill capacity constraints imposed on the nodes. If the former was not guaranteed, then the reward function would also need to check for whether there is any node that exceeds its capacity and return a negative or zero reward.

### 4.2. Reward Signal Approach

The reward we have chosen to maximize is the *average node occupancy ratio* (*O*). This value can be calculated by determining which are the active nodes (i.e., nodes that contain at least one task) and, then, averaging their occupancy ratio. The occupancy ratio of a given node is the sum of the cost of each of its allocated tasks divided by the capacity of the node. For example, the value of *O* in Figure 6 is 0.867 which is the mean of 1.0, 0.8 and 0.8.

It is noteworthy that other more direct metrics could also have been chosen. For instance, the inverse of the number of active nodes. However, the metric we have chosen is independent of the number of tasks in the input. This makes it possible for the agent to learn a policy that can be used with inputs having different numbers of tasks.

### 4.3. RL Method Used

It is not the objective of this work to present and explain all of the techniques and algorithms that fall under the RL umbrella. We will only explain the method used in our solution, which is a *DRL-based actor-critic policy gradient method*. The first reason why we chose this method over the rest was because it can handle very large (even infinite) state spaces, which is a requirement in our case (there are many possible sets of tasks). In addition, this method had shown fast convergence and good results for other types of combinatorial optimization problems [19].

In a DRL-based actor-critic policy gradient method, the policy is parameterized using a DNN. As mentioned in previous sections, the DNN maps each state to an action and we denote it by π(A∣S,θ). The symbol θ, as is common practice in ML and as explained in Section 2, represents the parameters of the DNN and stresses the fact that the mapping of states to actions depends on those parameters.

Before detailing the specific algorithm and formulas, let us explain the *training* process at a more conceptual level. Remember that we want to find the parameters θ for the DNN that maximize the reward. We start with a DNN with random values for θ. Policy gradient methods rely on running a large number of simulated interactions with the environment. A simulation starts by generating a random initial sequence of tasks $S_{t}$ (the state), we then use our existing DNN π(A∣S,θ) to generate a task allocation *A* (the action) and then we calculate the associated reward *O*. Lastly, the parameters θ of the DNN are then updated using a formula discussed in next paragraph. Each simulation and parameter update step is known as a *training step*.

Intuitively, if *A* has led to a *better reward than the average reward of the current policy π(A∣S,θ)*, we want to *update the parameters θ of the DNN in the “direction" that leads to more outputs like A*. In the formula that we present later in this section, the concept of “updating parameters in the direction that leads to more outputs like *A*” is represented by $\nabla_{\theta }p\left(\pi \left(A|S_{t},\theta_{t}\right)=A_{t}\right)$, which is the gradient with respect to the parameters θ of the probability of having chosen $A_{t}$. The concept of “better reward than the average reward that we were getting” is represented by the term $\left(O_{t}-\hat{v}\left(S_{t},\theta_{t+1}^{\prime }\right)\right)$, where $O_{t}$ is the obtained reward and $\hat{v}\left(S_{t},\theta_{t+1}^{\prime }\right)$ is a function that estimates the expected reward that will be obtained with the current policy and parameters $\hat{v}\left(S,\theta^{\prime }\right)$ given the sampled state $S_{t}$‘.

**Figure 7:**
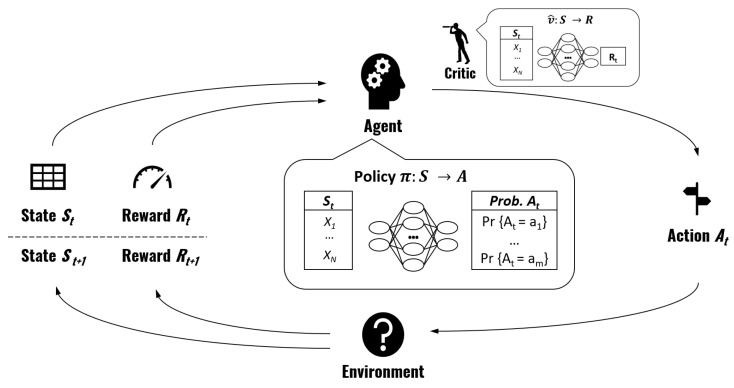
DRL-based actor-critic policy gradient. We use a DRL-based actor-critic policy gradient method. This means that two DNNs are trained. The first DNN parameterizes the policy used by the agent (a.k.a. actor) to map states to probabilities of taking each action. The second DNN parameterizes the critic, which maps states to the reward that it is expected to receive from the environment following the current policy.

The function $\hat{v}\left(S,\theta^{\prime }\right)$ is known as the *critic*. The critic is a *state-value* function, meaning that it takes a given state *S* and approximates the expected reward that will be obtained following policy $\pi_{\theta }$. We use another DNN with parameters $\theta^{\prime }$ to parameterize the critic. The critic parameters $\theta^{\prime }$ are also continuously updated during training. Figure 7 gives an overview of the training process.

The specific algorithm followed in the training process that we just described is the so-called *actor-critic reinforce stochastic gradient ascent algorithm*, based on the policy gradient theorem [17]. The algorithm (Algorithm 1), on each training step, updates the parameters θ and $\theta^{\prime }$ of the policy and the critic DNNs, respectively, as follows:
**Algorithm 1:** Algorithm used for the training
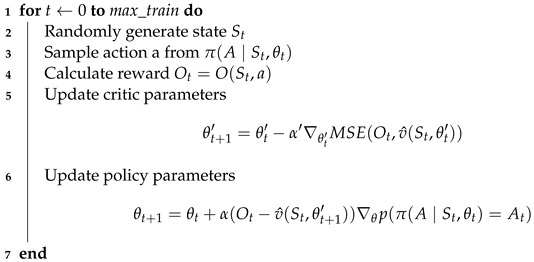


Note that the equation in line 5 is just a standard training step for a supervised ML regression problem where we want our critic DNN $\hat{v}\left(S,\theta^{\prime }\right)$ to learn to estimate O(A,S) and hence we use as a loss function the Mean Squared Error (MSE) of the difference between the obtained reward and the estimated one. $\alpha^{\prime }$ and α are the learning rates, which are simply two scalars representing the update “step size” for equations in lines 5 and 6, respectively. $p(\pi \left(A|S_{t},\theta_{t}\right)=A_{t})$ is the probability of having chosen $A_{t}$, given state $S_{t}$ and following policy $\pi (A|S_{t},\theta_{t})$. This probability can be calculated because the DNN that we use (more details on its architecture are provided in the next subsection) does not directly output a specific action (i.e., a specific mapping of tasks to nodes), but rather, it outputs a *probability for each possible action*. Therefore, we can know what the probability of choosing $A_{t}$ was.

A natural question is how we choose $A_{t}$ from all the possible actions. During training, we choose action $A_{t}$ by *sampling* it from the discrete probability distribution for each possible action given by our DNN π(A∣S,θ). Note that this is different to supervised ML, where we would select the action (or the class in supervised ML) with the highest probability. This means that during training we might select an action which does not have the highest probability according to policy π, but this is actually essential to ensure the *exploration* of the actions space, a fundamental principle in RL. During inference, however, once training has been completed, we make the policy *greedy* on the action values, meaning that in inference we always select the action with the highest probability.

### 4.4. Architecture of the Policy and Critic DNNs

We use a *pointer network* [22] to parameterize our policy π(A∣S,θ). A pointer network is a DNN architecture for solving variable length sequence-to-sequence problems and whose output is represented as a sequence “pointing” at positions in the input sequence. This is ideal for the input–output representation approach defined in Section 4.1.

The pointer-network follows an encoder–decoder Recurrent Neural Network (RNN) architecture [23]. This architecture is widely used in problems where the inputs and output are sequences (like our problem, but also in areas like Natural Language Processing since text is also sequential data), because it is designed to process each element of the input sequence *S* in order in what is known as the *encoding* (allowing the DNN to take the order of the elements into account) and then recurrently generate the output sequence *A* in what is known as the *decoding*. The decoding takes place in recurrent *decoding steps* where at each step *i*, the element *i* of sequence *A*, $a_{i}$, is a function of the input sequence *S* as well as of the previous outputs $a_{1},\cdots ,a_{i-1}$. We based our specific implementation on the architecture proposed by [19].

**Figure 8:**
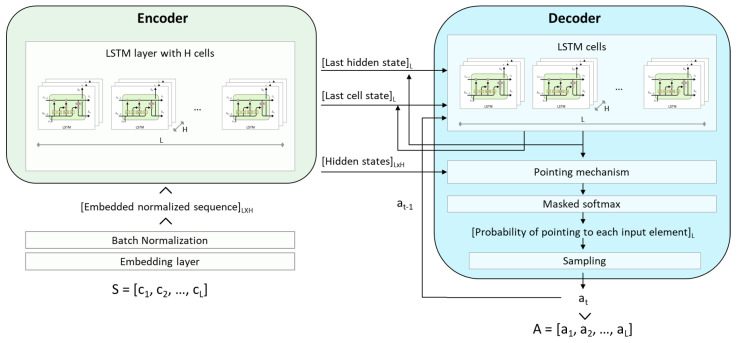
Neural network architecture. The encoder reads the input sequence $S=[c_{1},\cdots ,c_{L}]$ of task costs and ultimately produces an allocation order sequence *A*, which can be defined as numbers “pointing” at positions in the input sequence *S*.

See Figure 8 for a detailed overview of the architecture of our policy DNN. The encoder reads the input sequence $S=[c_{1},\cdots ,c_{L}]$ of task costs, one at a time, and transforms it into a sequence of latent memory states of dimension LxH. Where *H* is the hidden dimension of our DNN. Each element of the input sequence is first transformed into an H-dimensional embedding, obtained via a linear transformation shared across all input steps whose parameters are also learned. The decoder network also maintains its latent memory states and uses a *pointing mechanism* to produce a probability distribution over the next task that should be “pointed” at. Once the next task is selected (by sampling from the distribution), it is passed as the input to the next decoder step, together with the last memory states.

The pointing mechanism is based on the so-called Bahdanau’s attention mechanism first proposed in [24]. It takes the decoder hidden state as the query vector and the hidden states from the encoder as the reference vectors. The output of the pointing mechanism is masked first (to avoid pointing to the same input element twice) and then a soft-max activation is applied, so that the output can be interpreted as a probability distribution representing the degree to which the model is pointing to each element of the input sequence. The allocation order *A* (see Section 4.1) is generated by sampling $a_{i}$, at each decoding step *i*, from the probability distribution of $a_{i}$. The discrete density function of $a_{i}$ is the output of the DNN, which has a softmax activation in the last layer. Moreover, the results are appropriately processed before the softmax activation guarantees that the same position is not “pointed” twice.

The DNN used to approximate the critic function $\hat{v}\left(S,\theta^{\prime }\right)$ is made of an encoder with the exact same architecture as the DNN used for the policy, followed by a similar attention mechanism but using the encoder outputs as the query and reference vectors. Lastly, it has two standard feed-forward layers, the last one with a single neuron since $\hat{v}\left(S,\theta^{\prime }\right)$ is a regression model that attempts to estimate a scalar (i.e., the expected reward given state *S*).

### 4.5. Additional Remark for Readers Familiar with Other RL Problems

One thing to note is that we have defined an action as the sequence *A* representing the allocation order. This means that in our RL framework, when the agent receives a set of tasks that must be allocated, it allocates all of them in *one* action. Using the RL terminology: the *episode* lasts only one time step and the recurrent nature of the task allocation decision is already taken care of by the RNN architecture of the policy.

An alternative approach would have been to define an action as the allocation of a single task to a node and the RL episode would last *L* time steps, where *L* is the length of the input task sequence *S*. This alternative approach is equivalent to our approach if in the alternative one the reward is set to zero for all timesteps except for the last one, which would make sense because the average occupancy ratio is not known until the end (and using a partial average occupancy ratio on each timestep would cause the agent to deviate from learning to optimize the final average occupancy).

Our one-timestep approach is more convenient to implement. The only difference that needs to be taken into account is the equation in line 6 in Algorithm 1. In that equation, the term p(π(A∣S,θ)=a) represents the probability of having selected action *a*. In our approach *a* is actually a sequence *A* of *L* integers $a_{i}\in \left[1,L\right]$, i∈[1,L] (where $a_{i}$ represents the position of the task in the input sequence). Therefore, p(π(A∣S,θ)=a) in Equation (1) should be the probability of generating the sequence A following policy $\pi_{\theta }$, which can be calculated using the chain rule as:

(1) $$p\left(\pi \left(A|S,\theta \right)=\{a_{1},\cdots ,a_{L}\right\})=\prod_{i=1}^{L}p\left(\pi \left(a_{i}\right)|a_{1},\cdots ,a_{i-1},S,\theta \right)\}$$

$p(\pi \left(a_{i}\right)|a_{1},\cdots ,a_{i-1},S,\theta )$ is the probability of sampling $a_{i}$ in the decoding step *i* of the pointer network, which is given by the output of the masked soft-max layer (note that $p(\pi \left(a_{L}\right)|a_{1},\cdots ,a_{L-1},S,\theta )=1$ always because in the last timestep there is only a remaining task that can be pointed at).

## 5. Experiments

In this section, we show the experiments we have carried out to demonstrate how a DRL-based approach can achieve similar results to the best performing heuristic in terms of optimality of the allocation, while requiring less time to generate such allocation. The primary objective of our experiments is to evaluate how well the policy can learn to produce the allocation of tasks with high average occupancy *O*. In addition, we want to gain some basic understanding of the inference latency and of the model size, in line with our hypotheses presented in Section 3.5, related to the suitability of a DRL approach for time-sensitive and resource constrained environments like CADESs.

### 5.1. Experiments Approach

We have considered four different problem conditions (Table 1). In problems 1 to 3, the sets of tasks that the agent learns to allocate all have always the same size (24, 40 and 50, respectively). Problem 4 tests the ability of the agent to learn to allocate sets of tasks of variable length, with a minimum of 3 and a maximum of 50 tasks. This means that the same parameters θ of the policy DNN are applied to sequences of length 3, 4, 5, …, up to 50. In practice, all the sets of tasks are input as an array of length 50, but they might have zeroes at the end, meaning that there are no tasks. The DNN has two masking layers (one for the inputs and one for the outputs) that ensures these tasks are not taken into account.

We believe that these problem parameters represent quite well the conditions a real NM would have to face. The number of tasks proposed is one that we could find in real small/medium size CADES. The costs and capacities have been determined experimentally and we have paid some attention to choosing a set of conditions that did not easily lead to very high occupancy ratios, which could not pose a big enough “challenge” to our agent.

As discussed in Section 2, we assume that there is always at least one valid task allocation (i.e., there is always one valid allocation where no task is left unassigned). In our implementation, we enforce this by assuming that we always have the number of nodes required to fulfill the most optimal allocation. Therefore, the number of nodes available is not a fixed value which is part of the problem conditions, but rather we assume it is high enough to accommodate the generated task allocations.

We trained our parameterized policy to see how well it learns to allocate tasks, by analyzing the average occupancy ratio *O* achieved after 10,000 *training steps*. One training step consists of one iteration of the actor-critic reinforce stochastic gradient ascent algorithm described in Section 4.3.

As is common in ML, especially in stochastic gradient ascent algorithms like ours, we use *training batches*. This means that in each training step, we do not just use a *single* randomly generated state (i.e., a set of tasks), but rather, we use a *batch of randomly generated states*, which in our case is of size 128 (i.e., the batch consists of 128 randomly generated sets of tasks). In batch training, the parameters of the DNNs are updated using the mean of the individual updates calculated for each of the 128 samples (following equations in lines 5 and 6 in Algorithm 1). This approach, i.e., updating the parameters only once with the mean of the batch rather than 128 times with the value of each single element of the batch, has been shown to achieve the task more quickly and closer to the optimal convergence [16].

**Figure 9:**
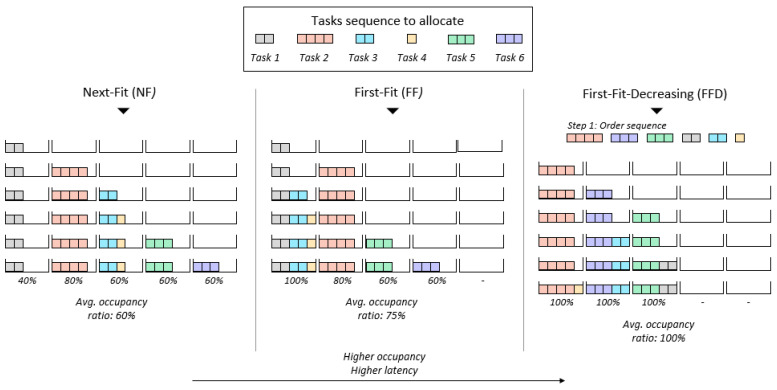
Benchmark heuristics. Visual example of how a given sequence of tasks would be allocated following each of the three benchmark heuristics.

To determine how well our solution works in comparison with popular heuristics [4] used to solve the bin-packing combinatorial optimization problem, we also solved the problems previously introduced using the Next-Fit (NF), the First-Fit (FF) and the First-Fit Decreasing (FFD) heuristics (see Figure 9). The NF and FF heuristics are the same as the ones described in Section 4.1, when discussing the input–output representation approach and the heuristic *H* assumed for the allocation order *A* generated by the DNN. Nevertheless, for ease of read, we describe the NF and FF heuristics again below, together with the additional heuristic used in the benchmark: the FFD. Thus, the benchmark heuristics are:

- *Next-Fit* (NF): Tasks in the input set are considered in the same order in which they were generated for the specific problem. They are sequentially allocated to the first “open” node until a task does not fit. When this occurs the node is “closed” and the next node is “opened”. A “closed” node cannot receive any additional task.
- *First-Fit* (FF): As with NF, the input is a set of tasks randomly generated. The only difference with the previous heuristic is that a node is not marked as “closed” unless it is completely full. Specifically, in each allocation step all the nodes are marked as “open” nodes. The task is allocated in the first node where it fits. If the task does not fit in any of the “opened” nodes, a new node one is “opened”.
- *First-Fit-Decreasing* (FFD): This heuristic is similar to the FF heuristic, but the input set of tasks is sorted, from the most costly to the least costly, before trying the allocation.

We selected the NF and FF heuristics because they are two well studied online heuristics used to solve this problem. Recall from the introduction that online means that they do not have to process the full input set prior to the start of the allocation. This type of heuristics is faster and has lower time complexity and lower memory requirements than offline heuristics such as FFD or our own DRL-based approach which, as opposed to online methods, require processing the entire set of tasks prior to generating a solution. That is precisely why we included two online heuristics in our benchmark. If such heuristics were to lead to a similar or better average occupancy ratio than our DRL-based approach, it would be hard to find reasons in favour of the DRL-based method. We also included an offline heuristic, FFD, in our benchmark because we also wanted to compare against a heuristic that is proven (and observed in our experiments) to yield results closer to the optimal than online heuristics. In order to calculate the average occupancy ratio obtained with each of the heuristics, we took the mean of 12,800 random samples of sets of tasks (100 batches of 128 samples).

As explained earlier, in our DRL approach, we take the allocation order *A* output by the DNN and test both the cases where tasks are allocated following an NF heuristic and following an FF heuristic. When looking at the average occupancy ratio that we obtain *just* using those heuristics, those are the baseline values for each respective case, since those are the average occupancy ratios that the policy would score if it does not manage to learn at all and simply generates random allocation orders *A*. We have not tried to improve the results with *hyper-parameter tuning*, i.e., changing different combinations of parameters in the network architecture and training an algorithm to see if it drives better results or with more sophisticated decoding strategies (e.g., beam search or those proposed by [19]), which could potentially yield better average occupancy ratio *O*.

In order to have it as a reference, we tried to calculate the optimal average occupancy for the four problems analyzed. We built our own iterative program and we even used Google’s tools for optimization [25]. However, it was not possible to solve problems with more than 15–20 tasks in a reasonable amount of time due to the amount of computation time required. For instance, for Problem 1 (which is the one with the shortest input length), it took the solver >10 h to find the solution for some of the samples. There are some exact algorithms such as in [14] that claim to be very fast for most of the problem instances, although there are still no guarantees of polynomial time. However, implementing such algorithms was beyond the scope of this work. It is worth mentioning that the optimal average occupancy is likely not far from the ratio obtained with the FFD heuristic: FFD guarantees a solution with no more than 11/9 of the optimal number of nodes. Refs. [4] and [14] empirically showed that the FFD achieved the optimal solution in 94.7% of the problem instances tested.

For the inference latency comparison, we will compare the time it takes to allocate the tasks following the FFD heuristic (the one that achieves better occupancy ratios) with the time it takes to generate an allocation with our agent, using the same hardware.

To understand the model size, we will just look at the storage space that the agent’s DNN uses in the hard drive, which is directly related to the number of parameters of the DNN and the format in which they are stored, which in our case is 32-bit integers.

### 5.2. Implementations of the Experiments

For each of the experiments, we trained three models, each with a random weight initialization, during 10,000 training steps and selected the model with the best results. An initial learning rate of 0.001 was used for both the agent and the critic network, with a 0.9 decay rate every 1000 steps. This simply means that the learning rate (i.e., the scalars $\alpha^{\prime }$ and α in equations in lines 5 and 6 in Algorithm 1) is reduced every 1000 steps by a factor of 0.9. This is shown to improve the convergence, because it helps to escape spurious local minima at the beginning of the training while avoiding oscillation around local minima at the end of the training [26].

The actor and critic DNNs were implemented in Python using the popular PyTorch framework [27]. We implemented both the DRL-based approach and the benchmark heuristics as functions in PyTorch and ran them on a computer with an Intel(R) Core(TM) i7-7600U CPU, no GPU and 32GB of RAM.

### 5.3. Experiment Results for the Performance

In Table 2, we show the average occupancy ratio obtained, in each problem, from our DRL agent and the selected heuristics. Our agent was able to match the best heuristic (the FFD) when being trained to generate allocation orders that then follow an FF rule. Perhaps more interesting, when trained to generate allocation orders that then follow an NF rule (which is faster than the FF and the FFD), it achieves a higher average occupancy ratio than the NF and the FF heuristics and gets close to the FFD performance. This is consistent across all experiments.

**Figure 10:**
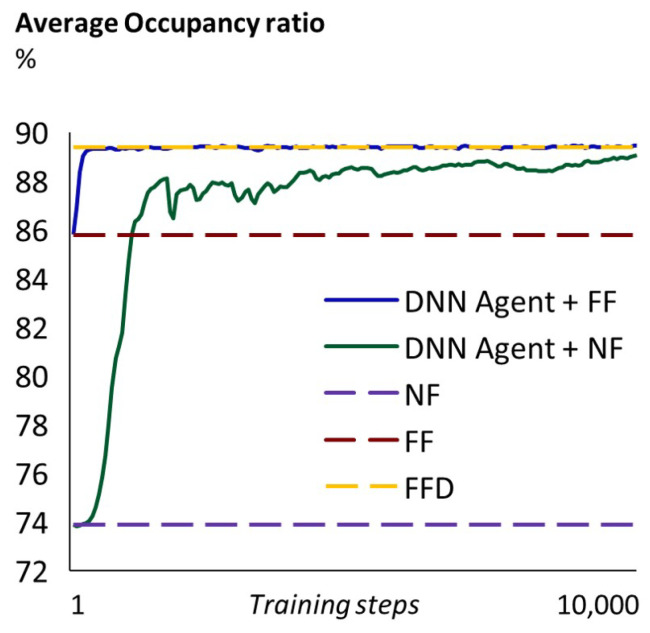
Training history. Problem 2: batch average occupancy ratio (%) after each training step, compared to the average occupation ratio obtained using the NF, FF and FFD heuristics.

In Figure 10 it can be seen how the average occupancy ratio that the agent achieves improves over time as the DNNs are trained (both the agent’s DNN and the critic’s DNN). When the agent uses FF, very good results can be obtained in a few hundreds of training steps. In contrast, when the agent uses NF, around 2000 training steps are needed to obtain better results than FF alone. After that point, various thousands of training steps are needed to get close to FFD. Note, however, that we can train offline the agent plus NF until it provides results that are very similar to FFD.

**Figure 11:**
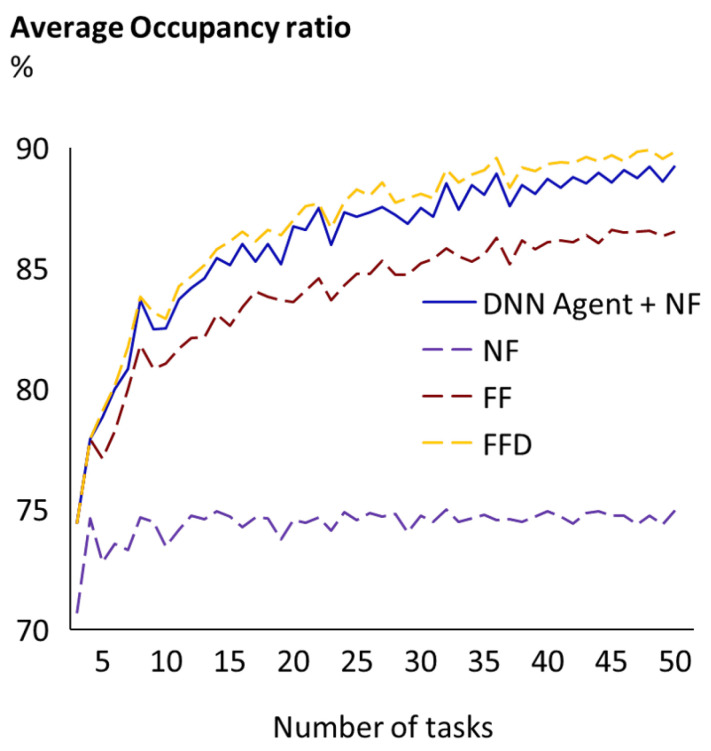
Problem 4 results by size of the input set of tasks. Average occupancy ratio obtained in Problem 4 when applying the trained DNN plus an NF heuristic and when applying the three benchmark heuristics.

For Problem 4, where the agent achieves an average occupancy ratio of 86.2% and 86.7% (depending on the allocation heuristic *H* used), we checked that results were consistent for all possible input lengths. As can be seen in Figure 11, the combination of the DNN plus the NF heuristic is consistently better than the NF and FF heuristics and consistently ends up close to the FFD for all algorithms. As expected, the average occupancy for all approaches tends to be higher the longer the sequence of tasks, because there are more possible combinations. The same consistency across all lengths is observed for the DNN plus the FF heuristic.

### 5.4. Experiment Results for Inference Latency

In this section, we describe the additional experiment we conducted to measure and compare the time required to generate a solution using our agent and the FFD heuristic. The problem conditions selected for this experiment cover many different sets of tasks with different sizes. The idea is to characterize the growth of the execution time as the problem becomes more complex. The specific problem conditions are shown in Table 3.

As concerns the heuristic used in conjunction with our agent, recall from the previous section that FF provides better results in terms of optimality than NF. However, it is possible to train the agent plus NF to achieve very similar results to the agent plus FF. Moreover, NF is faster that FF. Since in this test we are interested in the execution time, NF is the heuristic that is worth considering.

**Figure 12:**
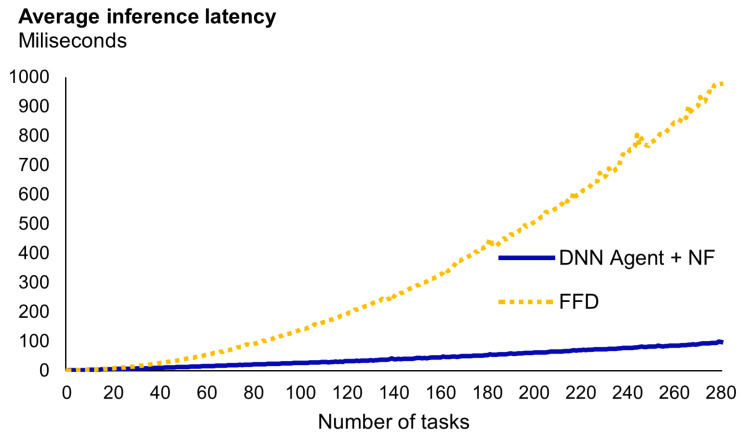
Problem 5 results by size of the input set of tasks.

In Figure 12, we show the results of the experiment. In this figure we can see that the time required for the FFD heuristic to determine the solution constantly increases as the number of tasks in the problem increases. This data corroborates what can be expected from the FFD heuristic, a temporal complexity of O(nlog(n)). In contrast, the inference latency of our approach exhibits a linear complexity, at least for these sets of tasks. In this regard, data corroborate that, when the size of the list of tasks is between 1 and 20 executions, execution times are alike. However, beyond 20, our approach is significantly faster than FFD. For instance, with around 30 tasks, FFD needs twice the time and for 50 tasks it needs triple the time.

One important aspect to highlight is that the values measured depend on implementation aspects such as the ML framework and the hardware used. For example, the use of GPUs, given their parallelization capacity, would speed up the DNN inference. However, the results obtained align with our initial hypothesis. A DRL-based solution can have a better time–reward trade off than heuristic-based approaches. This is especially notorious in devices with memory constraints, where sorting and searching algorithms cannot be implemented to minimize the response time.

Regarding the model size, our model required 264 KB of storage memory. The hidden dimension of our DNN (i.e., the number of neurons in the hidden layers) is 64. This is lower than typically used sizes (e.g., 128). However, having resource restrictions in mind, we wanted to check the performance of a lighter DNN. The pointer network architecture and the hidden dimension size of 64 resulted in the agent’s DNN having 66,754 parameters (this was for problem 3, where the input length was 50. The other problems had fewer parameters since the maximum length was smaller). These 66,754 parameters are ultimately stored as 32-bit integers requiring a storage memory of 264 KB. This is more than enough to fit on a standard microcontroller, which might have up to 1 MB of storage capacity [13].

## 6. Related Work

In this work, we use DNN to solve a combinational optimization problem. This is typically called *neural combinatorial optimization*. An important notable contribution in this regard was the pointer network architecture that we previously described. In [22], the authors prove that this type of architecture provides good results in combinatorial optimization tasks in the area of supervised ML.

A specific framework developed to address combinatorial optimization problems using DNNs together with RL was proposed by [19]. Our DNN architecture (except for the input and output layers which are specific to our problem’s input–output representation) and the selection of the specific RL algorithm to use (the actor-critic reinforce stochastic gradient ascent algorithm) is based on their work. However, they apply it to different combinatorial optimization problems with different problem conditions. We had to design all the aspects of this work that are specific to the task allocation problem (or equivalently, to the bin packing problem). For example, the input–output representation approach, the reward signal or the benchmark heuristics.

Taking the work in [19] as the starting point, multiple works have addressed the problem of allocation resources using DRL techniques. Some examples are [20,28,29,30]. Perhaps, the most related to ours are [20,30]. In [30], the authors propose a solution to allocate *services* to *hosts* while minimizing the power consumption. The reward function from this work is similar, but not identical, to the one we used. On the other hand, the architecture of the DNN, the problem constraints and the constraint-enforcing strategies are also different. In [20], the authors solve a packing problem in a 3D environment. The idea is to minimize the surface that is necessary to pack a set of items. This requires, for each problem instance, taking three decisions. In this work heuristics are used to take two of these decisions, while DRL is used for one of them.

To the best of the knowledge of the authors of the current work, this is the fist time DRL has been used to train a DNN to produce solutions that solve problems formulated as a 1D bin packing problem [4]. This is a problem that, although it can be simply formulated, is an NP-hard problem. Moreover, although here we study the allocation of tasks in CADESs, many workaday problems can be formulated and thus resolved using our solution. Finally, looking at the results obtained and, specifically, the level of performance in terms of optimality and the low response time indicates that the approach here described can be a better option than typical heuristics for implementing the NM in a CADES.

## 7. Conclusions and Further Work

In this work, we demonstrated that a solution to find task allocation in CADESs based on Machine Learning (ML) and on DRL in particular can achieve similar results to the best performing solutions based on heuristics in terms of optimality and the allocation, while requiring less time to generate such allocation.

Moreover, we fulfilled the specific objective of this work, which was stated as follows: design and implement an ML-based approach capable of allocating tasks to nodes in a CADES and analyze the benefits of such an approach both qualitatively and quantitatively. In Section 3, based on our research, we outlined, in a qualitative manner, the benefits of using a DRL-based approach in a CADES. With our solution design, implementation and experiments presented in Section 4 and Section 5, we have shown that:

- Our DRL agent, combined with an intermediate performing heuristic (the FF), can achieve average occupancy ratios similar to the best performing heuristic (the FFD).
- Our DRL agent can be used in combination with the fastest heuristic (the NF), yielding better average occupancy ratios than two popular heuristics (the NF and FF) and getting close to the best performing one (FFD).
- Our comparison in the response time supports the hypothesis that the inference latency of our agent can be significantly lower than the time required for the best performing heuristics to produce a solution. This is because the last ones can require pre-sorting the inputs.

Further work in this area could be targeted at modelling more complex operational contexts closer to real-life applications. On the one hand, this could be achieved by including a wider range of variables in the state information consumed by the DRL agent. Examples of these additional variables are the time required by each task to complete its execution, the number of nodes available (which could change over time due to node failures or incorporations) or a varying computational capacity for the nodes (which could vary over time due to, for example, some nodes being partially busy executing a task that cannot be reallocated). On the other hand, more complex operational contexts could also be factored in the reward function that defines the allocation criteria. For instance, the cost of reallocating a task could be factored in the reward function, which would give priority to task allocations that do not require moving a large number of tasks among nodes.

We are convinced that the full potential of DRL-based configuration search can be realized when modelling more complex operational contexts such as the ones just described. The more complex the problem is, the harder it is to find heuristics or search strategies that deliver good results in a timely manner and that is precisely where a versatile approach such as using a DRL-trained policy can have a greater impact.

## Figures and Tables

**Table 1:** Problem conditions in our experiments. Task costs are sampled from the uniform distribution over the interval [Min. task cost, Max. task cost].

Problem Label	# of Tasks	Min. Task Cost	Max. Task Cost	Capacity
1	24	1	6	8
2	40	3	8	10
3	50	4	14	15
4	Variable (3 to 50)	4	14	15

**Table 2:** Optimization performance experiments results. Avg. occupancy ratio (%) comparison between our trained DRL agents and the selected heuristics.

Problem Label	Average Occupancy Ratio %				
	DRL Agent		Heuristics		
	DNN + NF	DNN + FF	NF	FF	FFD
Problem 1	92.3	93.8	77.5	89.2	93.8
Problem 2	89.1	89.4	73.9	85.8	89.4
Problem 3	89.3	89.8	74.7	86.5	89.8
Problem 4	86.2	86.7	74.3	84.4	86.7

**Table 3:** Problem conditions in our 5th experiment. Task costs are sampled from the uniform distribution over the interval [Min. task cost, Max. task cost].

Problem Label	# of Tasks	Min. Task Cost	Max. Task Cost	Capacity
5	1 to 280	1	50	50

## Data Availability

Not applicable.
